# The best treatment option(s) for adult and elderly patients with chronic primary musculoskeletal pain: a protocol for a systematic review and network meta-analysis

**DOI:** 10.1186/s13643-019-1174-6

**Published:** 2019-11-09

**Authors:** Helen Koechlin, Ben Whalley, Nicky J. Welton, Cosima Locher

**Affiliations:** 1 Division of Clinical Psychology and Psychotherapy, Faculty of Psychology, University of Basel, Basel, Switzerland; 2 Department of Anesthesiology, Critical Care, and Pain Medicine, Boston Children’s Hospital, Harvard Medical School, Boston, MA USA; 3 School of Psychology, University of Plymouth, Portland Square, Plymouth, PL4 8AA UK; 4 Population Health Sciences, Bristol Medical School, University of Bristol, Bristol, UK

**Keywords:** Chronic primary musculoskeletal pain, Network meta-analysis, Adults, Elderly, Intervention

## Abstract

**Background:**

Chronic primary musculoskeletal pain (CPMP) is one subcategory of the new classification of chronic primary pain for the upcoming ICD-11, defined as chronic pain in the muscles, bones, joints, or tendons that persists or recurs for more than 3 months and is associated with significant emotional distress or functional disability. An array of pharmacological, psychological, physical, complementary, and rehabilitative interventions is available for CPMP, for which previous research has demonstrated varying effect sizes with regard to effectiveness in pain reduction and other main outcomes. This highlights the need for the synthesis of all available evidence. The proposed network meta-analysis will compare all available interventions for CPMP to determine the best treatment option(s) with a focus on efficacy and safety of interventions.

**Methods:**

We are interested in comparing interventions of the following types: psychological, pharmacological, physical, complementary, and rehabilitative interventions. We will include all randomized controlled trials that compare one intervention with another, or with a control group, in the treatment of CPMP. Primary efficacy outcomes will be pain intensity, emotional distress, and functional disability. Safety outcomes extracted will include proportion of patients with treatment-emergent adverse events, unwanted events, or drop-out rates due to side effects. Published and unpublished trials will be sought through the search of all relevant databases and trial registries. At least two independent reviewers of the team will select the references and extract data independently. We will assess the risk of bias of each individual study using the Cochrane risk of bias assessment tool. We will conduct a network meta-analysis to synthesize all evidence for each outcome. We will fit our model primarily within a Bayesian framework.

**Discussion:**

CPMP is a disabling condition for which several interventions exist. To our knowledge, this is the first network meta-analysis to systematically compare all available evidence. This is required by national health institutions to inform their decisions about the best available treatment option(s) with regard to efficacy and safety outcomes.

**Systematic review registration:**

PROSPERO CRD42018096114

## Background

In 2015, an International Association for the Study of Pain (IASP) Task Force, comprised of pain experts from across the globe, suggested a new and pragmatic classification of chronic pain for the upcoming ICD-11. This classification includes seven categories, among them chronic primary pain (CPP). This new definition was created because the etiology for most pain conditions is unknown; CPP describes a condition where *pain itself* is the disease [1]. CPP is defined as pain in one or more bodily regions that persists or recurs for more than 3 months, is associated with significant emotional distress or functional disability, and is not better explained by another chronic pain condition [2]. CPP consists of the following subcategories: chronic widespread pain, complex regional pain syndrome, chronic primary headache and orofacial pain, and chronic primary visceral pain, as well as chronic primary musculoskeletal pain [2]. These different categories share the following multiple similarities that allow to summarize them under the umbrella term CPP [3–5]: (1) high comorbidity rates between chronic pain conditions [6], (2) similar interventions across syndromes [7], (3) similar psychiatric comorbidities (i.e., mainly anxiety and depression) [8], (4) similar societal challenges (e.g., sick days and use of health care services) [9], and (5) the syndromes share genetic factors [10].

The ICD-11 defines chronic primary musculoskeletal pain (CPMP) as chronic pain in the muscles, bones, joints, or tendons that is characterized by significant emotional distress (i.e., anxiety, anger, frustration, and depressed mood) or functional disability. CPMP poses a major problem of public health, considering its high prevalence and refractory nature [11]. Low back pain and neck pain contribute to 1694 years lost to disability per 100,000 persons annually. The two disorders rank first and fourth as global causes of years lived with disability [12]. These conditions also reduce quality of life by interfering with work [11]. From a societal perspective, chronic musculoskeletal pain creates substantial costs for healthcare systems, disability insurance, and work absenteeism [13].

Interventions for CPMP include pharmacotherapy, psychotherapeutic interventions, physiological therapies, rehabilitative interventions, and complementary medicine. Pharmacotherapy plays an important role in alleviating pain for these patients. Non-steroidal anti-inflammatory drugs (NSAIDs), muscle relaxants, and opioid analgesics are some of the most common classes of drugs provided [14]. With regard to effectiveness, pharmacological interventions show small to moderate effect sizes in recent meta-analyses compared to placebo [14, 15]. For example, Gabapentinoids showed minimal improvement of pain when compared to placebo [16], and opioids showed better pain reduction for chronic low back pain than placebo, with standardized mean differences (SMDs) ranging from − 0.55 for tramadol to − 2.47 for transdermal buprenorphine, and strong opioids showed an SMD of − 0.43 [17]. A network meta-analysis looking at paracetamol, NSAIDs, and opioid analgesics is underway [18]. Additionally, vitamin D supplementation recently showed no effect on pain (SMD = 0.004) [19]. It has been argued that the small effects might be attributed to the choice of the primary outcome measure (i.e., pain reduction) in pharmacological trials [20]; other outcomes such as quality of life might show greater gains.

Non-pharmacological interventions also reveal small to moderate effect sizes. For example, psychological interventions such as CBT and self-regulatory treatments proved superior to several control groups for pain intensity (SMD = 0.41), pain interference (SMD = 0.23), and quality of life (SMD = 0.41) at post-treatment [21]. Along similar lines, a recent meta-analysis reported that a self-management group for patients suffering from chronic musculoskeletal pain revealed a moderate, significant effect size (SMD = 0.28) when compared to a range of control groups (such as waitlist control, treatment as usual, exercise, and education); however, the difference was not clinically significant [22]. Other meta-analyses in the field of self-management training [23], multidisciplinary biopsychosocial rehabilitation programs [24], and pain education approaches [25] draw similar conclusions. For physiological and alternative approaches, it has been shown that Tai Chi is more effective than no treatment or usual care in reducing pain in the short-term (SMD = 0.66) [26]. Comparable effects have been found for exercise interventions [27], whereas no significant differences in pain were found between a Pilates and a control group [28]. In terms of complementary approaches, patients suffering from nonspecific chronic low back pain reported a clinically meaningful reduction in levels of pain when participating in an acupuncture group [29]. However, it should be noted that the pharmacological and non-pharmacological studies did not use similar follow-up periods. Drug studies usually have shorter timeframes than psychological ones, and a direct comparison of the results is not advisable.

Besides effectiveness, the safety of a specific intervention informs clinicians and guidelines. For example, Tapentadol has shown to be associated with a 2.7-fold increase in the risk of discontinuation treatment due to adverse effects [15]. Non-pharmacological interventions may also cause unwanted events but are, however, less frequent and less likely to be reported. A meta-analysis of multicomponent treatments (i.e., those that involve a combination of physical, psychological, educational, and/or work-related components) looked at adverse events, but the included studies reported adverse events in a manner that did not enable comparisons between groups [24]. Other available meta-analyses in the field of CPMP did not report safety outcomes, and to date, no network meta-analysis is available (except for one protocol [18] that looks at paracetamol, NSAIDs, and opioid analgesics for chronic low back pain).

Aging and disability are both related to an increased occurrence of chronic pain [30]. Despite increasing awareness of the prevalence of pain among older adults, under-treatment remains an issue, not only because of polypharmacy and comorbid diseases, but also because of commonly held pain myths which are assumed to complicate care in the elderly [31]. To the best of our knowledge, meta-analyses focusing on this vulnerable age category are scarce, with the exception of one recent meta-analysis, examining exercise interventions in elderly patients suffering from chronic musculoskeletal pain [32].

Previous research on efficacy and safety data has predominantly focused on the comparison of one medication versus a control group or has applied a comparative design (e.g., the effectiveness and safety of one medication compared to another) [18]. However, from the perspective of patients and doctors selecting treatments, it would be preferable to compare multiple interventions simultaneously, and to quantify the likely range of outcomes when selective between the highly diverse interventions available. Here, the proposed network meta-analysis uniquely allows to address these points.

## Objectives

The objective of this systematic review and network meta-analysis (NMA) is to synthesize all available data from RCTs on interventions delivered for CPMP in adult patients, including the elderly. Our aims are to (1) compare the efficacy (i.e., changes in pain, emotional distress, and functional disability) and (II) safety (i.e., treatment-emergent adverse events, serious adverse events, unwanted events) of all available interventions with each other and various control groups. We will focus on post-treatment assessment, medium-term, as well as long-term follow-up, whereby long-term outcomes will be considered primary. Our analysis will incorporate information on the relative efficacy of control interventions used within included studies, and, where feasible, we will include control conditions within the matrix of treatment comparisons (e.g., treatment-as-usual) [33].

## Methods and analysis

### Protocol and registration

Our protocol is registered on PROSPERO (CRD42018096114) and is available from http://www.crd.york.ac.uk/PROSPERO.

### Eligibility criteria for considering studies for review

#### Types of studies

We will include RCTs that make head-to-head comparisons of two different interventions as well as those that compare an intervention with a control group (e.g., waitlist control, treatment as usual, or placebo) in the treatment of CPMP. We will include protocols and conference papers. Cross-over trials will be included if the results of the first administration are reported separately. Further, randomized single control studies (e.g., the comparison of different dosages of one intervention) will be included. Finally, prophylactic interventions will be included and form a separate network as long as they target CPMP.

We will not include case-control studies, post hoc analyses, or secondary analyses (for example those reporting the cost-effectiveness of an intervention where the primary study focused on the efficacy of the intervention).

#### Types of participants

Adult patients, including the elderly, of both sexes, with a primary diagnosis that can now be classified as CPMP [2] will be included. We will classify chronic pain syndromes as CPMP according to the 11th revision of the International Classification of Diseases (ICD-11). Hence, the syndromes to be included are now established as chronic low back pain, chronic cervical pain, chronic thoracic pain, and chronic limb pain. In line with the ICD-11, we will only include patients suffering from pain that occurs in one or more bodily regions and that persists or recurs for more than 3 months. Also, the diagnosis of CPMP is appropriate independently of identified biological or psychological contributors unless another diagnosis would better account for the presenting symptoms. Other ICD-11 chronic pain diagnoses, namely chronic cancer-related pain, chronic postsurgical or posttraumatic pain, chronic neuropathic pain, chronic secondary headache or orofacial pain, chronic secondary visceral pain, and chronic secondary musculoskeletal pain, will be excluded. Additionally, according to the ICD-11, CPMP is characterized by significant emotional distress or functional disability. However, the studies we include will rely on older diagnostic criteria of chronic pain (e.g., DSM-III, DSM-III-R, DSM-IV, DSM-5, and ICD-10). Therefore, we will also include patients suffering from chronic pain where no additional information regarding their emotional or functional disability is provided.

Studies in which patients have a diagnosis of acute pain (MG31), headache disorders (8A80-8A8Z), or a chronic primary pain syndrome other than CPMP will not be included. Also, studies reporting on chronic secondary pain (i.e., where pain is a symptom of an underlying condition) will be excluded.

#### Types of interventions

We will include all available interventions for CPMP. This includes (but is not limited to) psychological interventions such as CBT, pharmacological agents such as opioid and non-opioid analgesics, physical interventions such as exercise therapy, complementary interventions such as acupuncture, and rehabilitative interventions such as multidisciplinary inpatient treatment. We will not only focus on face-to-face interventions, but also e-health and m-health approaches. We do not require the treatment to have a minimal length or to be provided by a trained clinician. See the “Data extraction” section for more details on what will be extracted from individual studies.

### Efficacy outcome measures

For each efficacy outcome, we plan to conduct a separate NMA.

#### Primary outcomes

The choice of our primary outcomes is in line with recommendations for clinical trials studying chronic pain (IMMPACT initiative) [34] as well as recent (network) meta-analyses and reviews [15, 18, 35, 36] in the field. Furthermore, we decided to focus on self-reported measures of pain rather than on clinical measurements (e.g., walking test) or objective measures (e.g., measure of pain threshold) since first, self-report measures provide the “gold standard” in the assessment of pain outcomes, and second, to acknowledge the inherently subjective nature of pain. Finally, we intend to prioritize global scores over syndrome-specific scores since the definition of CPMP includes various syndromes [37].

Global pain intensity and the global measurement of pain will be our primary outcomes. We will extract both outcomes where both are reported. We plan to model them jointly, because we assume that these two measurements correspond to the same underlying construct, and relative intervention effects on each will be correlated. We will conduct an additional sensitivity analysis in which these outcomes will be analyzed separately.

1. Global pain intensity. Pain intensity may be measured with a continuous self-report scale (e.g., visual analog scale (VAS) or numeric rating scale (NRS)), or an ordinal scale with greater than six levels (we will consider such ordinal scales to exhibit continuous properties). It should be noted that for the majority of clinical trials of CPP treatments, a measure of pain intensity will provide the primary outcome measure [34].
2. Global measurement of pain. A rating scale within a composite measure of pain (e.g., Short-Form McGill Pain Questionnaire (SF-MPQ) [38]).

In line with the core definition of CPMP (i.e., significant emotional distress or functional disability), we decided to define emotional distress as well as functional disability as additional primary outcomes of interest. Furthermore, the evaluation of additional symptoms other than pain is clinically important since studies reveal that pain intensity and (physical) functioning are only modestly associated [39].

3. Emotional distress. Emotional distress includes symptoms such as depression, anxiety, and anger in CPMP patients [40]. Questionnaires such as the Beck Depression Inventory (BDI) [33], the Hospital Anxiety and Depression Scale (HADS) [41], and the Profile of Mood States (POMS) [42] will be extracted.
4. Generic measures of functional disability. Measurements of the interference of pain with physical functioning. Use of global questionnaires such as the Multidimensional Pain Inventory (MPI) [43] as well as the Interference Scale or the Brief Pain Inventory (BPI) [44, 45] with pain interference items (i.e., general activity, mood, walking ability, work, relations with other people, sleep, enjoyment of life) will be extracted.

#### Secondary outcomes

The secondary outcomes are as follows:

1. Syndrome-specific pain. Self-rated questionnaires which specifically measure pain in the field of CPMP (e.g., the Aberdeen Back Pain Scale, ABPS for chronic low back pain) [46].
2. Syndrome-specific measures of physical functioning. Self-rated questionnaires which specifically evaluate physical functioning for patients suffering from chronic musculoskeletal pain (e.g., Roland and Morris Back Pain Disability Scale for chronic low back pain) [47].
3. Health-related quality of life (HRQOL). The SF-36 Health Survey [48] is the most commonly used generic measure of HRQOL. However, we will also extract other questionnaires such as the EuroQol EQ-5D [49].
4. Participant ratings of global improvement and satisfaction with treatment. This category will evaluate all aspects of patients’ health and assesses if there has been an improvement or decline in clinical status (e.g., Patient Global Impression of Change scale, PGIC) [50].

### Safety outcome measures

For each safety outcome, we plan to conduct a separate NMA.

Besides efficacy outcomes, we are also interested in safety outcomes, including treatment-emergent adverse events (TEAEs; e.g., dizziness and nausea), serious adverse events (SAEs; e.g., increased risk of suicidal thoughts and behavior), and other unwanted events (UEs). Adverse effects are described broadly as any of “adverse event,” “adverse drug reaction,” “side effect,” “toxic effect,” “complication,” and “unwanted events” that are associated with the treatment under investigation (may it be pharmacotherapy, psychotherapeutic interventions, complementary medicine, physiological therapies, or rehabilitative interventions). We will also extract the length of the treatment period for each study in order to calculate the results for the safety outcomes. We will evaluate three different reporting methods of safety outcomes:

1. We will extract the proportion of patients who experience TEAEs, SAEs, or UEs during the treatment period (i.e., the latest reported time point).
2. We will also extract the mean number of TEAEs, SAEs, or UEs per patient across all reported symptoms (i.e., for the latest reported time point).
3. Finally, study discontinuation due to TEAEs, SAEs, or UEs is also defined as an additional safety outcome measure.

### Search strategy and study selection

Searches for published RCTs will be undertaken in the following electronic databases: MEDLINE, Embase, PsycINFO, Cochrane Central, and Web of Science. A first search has been conducted until April 5th, 2018, and will be updated in October 2019. In addition to that, we will search for unpublished RCTs at clinicaltrials.gov and OpenTrials. It is important to include unpublished data, since publication bias leads to exaggerated effect sizes [51] and reporting bias can bias NMA-based estimates of treatment efficacy and modify ranking of interventions [52]. Studies will be identified using search terms for chronic pain in general (*chronic pain*) and specific syndromes (such as *fibromyalgia*, *complex regional pain syndromes*, and *irritable bowel syndrome*). See Additional file 1 for the search strategy. Note that the search strategy also includes all other categories of chronic primary pain (i.e., chronic widespread pain, complex regional pain syndrome, chronic primary headache and orofacial pain, and chronic primary visceral pain). The reasons for this are twofold: first, we wanted to make sure that our search is exhaustive and second, the long-term goal of this project is to build up a database on all existing interventions for chronic primary pain syndromes. Also, due to the high number of search returns, each individual chronic primary pain category will be reported separately. No data limits or language restrictions will be applied to any of the searches. Results will be exported into Covidence, a web-based software by Cochrane to assist with the production of systematic reviews (https://www.covidence.org).

A research team (i.e., around 15 people) will independently review references and abstracts retrieved by the search and exported into Covidence (https://www.covidence.org/home). The team will have a regular exchange (i.e., once in a month) in order to clarify uncertainties and questions related to the screening process. Each paper will be evaluated by two raters independently. Conflicts will be resolved by the two primary investigators (CL and HK). Consensus will be reached by a weekly consensus meeting of the two primary investigators. If no consensus can be reached, an independent expert in the field of CPP will be contacted. We will obtain the full text of all articles included by reference and abstract screening and use the same eligibility criteria to determine inclusion or exclusion. In case of missing information on these domains in the article, the trial authors may be contacted to obtain further information.

### Data extraction

Each article/study-report will be read, evaluated for completeness of the data and be rated for quality by two independent reviewers (for quality ratings, see details below). A structured data extraction form will be designed and pilot-tested for 30 randomly selected studies. Information extracted will include *study design* (type of study such as parallel or cross-over design, blinding of treatment, special inclusion criteria, special population [if 80% or more of the sample share a particular characteristic]), *study characteristics* (such as lead author, publication year, journal, sponsor, region and setting, number of clinical sites), *participant characteristics* (such as diagnostic criteria, age, sex, pain syndrome, pain intensity, comorbidities, duration of symptoms, age of onset), *intervention details* (such as type of intervention, drug dose and dosing schedule [fixed vs. flexible], length of intervention, training of provider, co-intervention/prophylaxis), *control group details* (such as type of control group, placebo lead-in yes/no, control dose and dosing schedule, length of control), *analysis details* (such as intent-to-treat, secondary or sub-analysis), and *outcome measures* (see the “Outcome measurements” section). After the extraction, we will carefully check and decide which demographics will be used for moderator analyses.

#### Outcome measurements

We will extract means (Ms) and standard deviations (SDs) for our continuous outcomes (i.e., efficacy outcomes). We will also extract the number of events (*E*) as well as the total occurrences (N) for each trial arm for our categorical outcomes (i.e., safety outcomes). We will extract data in a wide format (i.e., single study per row) for both continuous and categorical outcomes (i.e., efficacy and safety outcomes). Two review authors (HK and CL) will ascertain that the data are entered correctly into the final data set. We will evaluate whether published and unpublished studies provide different values, using sensitivity analysis.

#### Missing outcome data

If SDs are not provided for the continuous outcomes, we will calculate them from standard errors (SEs), confidence intervals [53] or other measures [54, 55]. If we are unable to calculate SDs, we will impute the mean of SDs from studies using the same outcome measure [56]. It should be noted that the SDs depend on the scale of measurement which can vary between studies even for the same outcome measure (e.g., VAS for global pain intensity ranging from 1 to 10 or from 1 to 100). Therefore, we will adapt the imputation procedure: SDs vs. Ms will be plotted across all studies where SDs are provided. This will allow us to use a regression model to more precisely predict the SDs in studies without them, based on the reported mean in that particular study. We will conduct an additional sensitivity analysis (all studies, including those with imputed SDs vs. only those studies that report SDs). If the sensitivity analysis reveals a significant difference, we will exclude studies with missing SDs. If the *N* is missing in the table of analysis, we will use the *N* of the descriptive statistics (e.g., patients randomized). In the absence of continuous outcome data, we will extract the number of patients that fulfilled a certain criterion (e.g., pain relief) and the number of patients that did not fulfill the respective criterion [57].

#### Unit of analysis issues

For cross-over studies, only the first period before the cross will be extracted [58]. In the case of cluster-randomized trials, we will extract data that takes the clustering into account (i.e., adjusted results). If such adjusted data are not available, we will extract unadjusted results and will adjust the sample size by dividing it with the design effect.

#### Length of trial

In line with other meta-analytical approaches [24, 59], we will extract outcome assessment data for a maximum of three time periods and group them for the purposes of disseminating the analyses: *post*-*treatment assessment* (i.e., measured at the time point closest to the end of treatment), *medium*-*term* (i.e., at least 3 months but less than 12 months), and *long*-*term follow*-*up* (i.e., 12 months or more) measured from end of treatment. We considered long-term outcomes to be primary.

### Risk of bias in individual studies

We will assess risk of bias in the included studies using the revised Cochrane risk of bias assessment tool [60]. The assessment will be performed by two raters independently. Any disagreements will be resolved by the primary investigators (HK and CL). The risk of bias will be evaluated across the following domains: generation of allocation sequence, allocation concealment, blinding of study personnel and participants, blinding of outcome assessor, attrition, selective outcome reporting, and other domains, including sponsorship bias. We will conduct a sensitivity analysis and compare all studies vs. only those studies with low or moderate risk of bias (i.e., excluding those with high risk of bias). We will pilot-test the risk of bias assessment procedure on a small number of studies.

### Statistical synthesis of study data

#### Characteristics of included studies and information flow in the network

We will present descriptive statistics for each trial, including *study characteristics* (e.g., lead author, publication year, journal, sponsor, region and setting, number of clinical sites) as well as *participant characteristics* (e.g., diagnostic criteria, age, sex, pain syndrome, pain intensity, comorbidities, duration of symptoms, age of onset). We will also describe the types of comparisons. The available results will be presented in a network diagram for each outcome. The size of the nodes will reflect the amount of evidence accumulated for each treatment (total number of patients), the breadth of each edge will be proportional to the inverse of the variance of the summary effect of each direct treatment comparison, and the color of each edge will represent the risk of bias assessment.

#### Pairwise meta-analyses

For the pairwise meta-analyses, mean differences of efficacy outcomes (e.g., global pain intensity) between treatment groups will be estimated for each study and will be pooled using a standardized mean difference (SMD). In the case where we have pre- and post-measures only and we want to calculate the standard error (SE) for change from baseline, a correlation coefficient (*r*) is needed. In a first step, we will set *r* = 0.5. However, we will conduct additional sensitivity analyses, defining *r* = {0.3, 0.7} [56]. For our safety outcome measures, we will calculate odds ratios (ORs) with 95% credible intervals.

Heterogeneity will be assessed by calculating the *Q* statistic, the *τ*^2^, and the *I*^2^, a transformation of *Q* that indicates the proportion of observed variance that can be attributed to heterogeneity rather than sampling error [61, 62]. However, we will prefer *Q* statistic and the *τ*^2^ over the *I*^2^, as the *I*^2^ is sensitive to sample size [63].

#### Network meta-analyses

For the network meta-analyses of efficacy and safety outcomes, we will employ Bayesian methods, using JAGS via R [64] using the R package gemtc (https://cran.r-project.org/web/packages/gemtc/gemtc.pdf). Initially, we will set broad, pessimistic, weakly informative priors for treatment effects [65]. With regard to the nodes in the network, we plan to start by splitting the interventions and plot networks in a first step. However, this might lead to unconnected networks. Therefore, in a second step, we will discuss ways to classify the interventions into groups, relying on clinical experts in this area to gain an understanding of the different interventions. Other aspects such as length of sessions and training of treatment providers will be included as regression covariates. In a sensitivity analysis, we will contrast this with models using priors elicited from the study team and our clinical colleagues.

We will calculate SMDs for our continuous efficacy outcomes and risk (i.e., probability of study discontinuation due to side effects) or rate ratios (i.e., proportion of patients who experience TEAEs or SAEs and the mean number of TEAEs and SAEs across all participants) for our categorical safety outcomes. We will present the summary SMD as well as ORs for all pairwise comparisons in a league table. We will use random-effects models rather than fixed-effects models because we expect that the included studies will be heterogeneous [66]. However, we will assess heterogeneity and if there is no evidence of heterogeneity, then a fixed-effects model will be more appropriate.

In case of an unconnected network (e.g., two separate networks for pharmacological and psychological interventions), we plan to conduct and report results from each of the networks analyzed separately. We will also use these statistical models to summarize likely efficacy outcomes (and uncertainty in those outcomes) from the perspective of a patient choosing between interventions.

#### Assessment of the transitivity assumption

The transitivity assumption states that we can combine the direct evidence from AC and BC studies to learn (indirectly) about the comparison AB [67]. We will evaluate the assumption of the transitivity by epidemiologic judgment considering three of its “equivalent expressions” by Salanti [68, 69]: (1) whether studies are comparable in terms of the distribution of effect modifiers, (2) whether the direct and indirect treatment effects are in statistical agreement (via an assessment for inconsistency), and (3) whether participants included in the network could in principle be randomized to any of the treatments.

#### Subgroup analyses

We will test whether some interventions have treatment effects dependent on what syndrome and age group (i.e., adults vs. the elderly) they are being applied in. This will add an extra level of hierarchy.

#### Sensitivity analyses

Multiple sensitivity analyses are planned and described in the according paragraphs. In short, these include (1) double-blind vs. unblinded trials (this is especially important for pharmacological interventions since there, double-blinding is possible and preferable), (2) global pain intensity vs. global measurement of pain (we will analyze them separately in this sensitivity analysis in order to justify the pooling across the two outcomes), (3) published vs. unpublished, (4) imputed vs. reported SD, (5) high risk of bias vs. low risk of bias, and (6) different levels of correlation coefficients where SE for change from baseline has to be calculated (*r* = 0.5 vs. *r* = {0.3, 0.7}).

#### Assessment of inconsistency

Transitivity will be tested with a statistical evaluation of consistency, i.e., the agreement between direct and indirect estimates. We will apply local as well as global methods to detect inconsistency [67, 70, 71]. Local methods focus on the inconsistency of a specific treatment comparison, whereas global methods check for inconsistency in the entire network of evidence [67, 71]. We will apply a local method to test for incoherence, namely node-splitting, which compares direct and indirect relative treatment effects using a *Z*-test [71] and a *p* value of test for disagreement (direct versus indirect evidence). In order to calculate node-splitting, we will use the R package gemtc. We will also evaluate consistency in the entire network by calculating the unrelated mean effects (UME) model [71]. In this model, the consistency equations are completely removed, which is equivalent to a series of independent meta-analyses for each pairwise contrast while sharing a common heterogeneity variance. The fit of this model is then compared with the standard consistency NMA model [67]. It should be noted that for both local and global approaches, it will only be possible to assess inconsistency if the network presents with closed loops.

Tests for inconsistency are known to have low power [53, 72], and it has been reported that 10% of evidence loops are expected to be inconsistent in the medical literature [73]. Accordingly, we plan to interpret the statistical inference regarding inconsistency with caution.

#### Selection bias

We will apply the comparison-adjusted [53] as well as the contour-enhanced [74] funnel plots in order to investigate whether the results in imprecise trials significantly differ from those in more precise trials. Additionally, we will perform a meta-regression with a measure of study precision as a covariate, which allows us to explore if there are issues with small study effects [75].

## Discussion

The introduction of the new CPMP classification in the ICD-11 both enables and requires the generation of new evidence regarding its treatment options. The network meta-analytic approach will synthesize already existing syndrome-specific evidence in a single network, resulting in efficacy and safety outcomes of multiple interventions for CPMP. Also, evidence gaps in the new field of CPMP will be identified.

National organizations such as the National Institute for Health and Care Excellence require the synthesis of all available evidence from existing studies to inform their decisions about the best available treatment option(s) with regard to safety and efficacy outcomes [76]. The planned network meta-analysis will provide these organizations, clinicians, and patients with this information, potentially helping to inform clinical guidelines.

## Supplementary information

**Additional file 1:** Search strategy.

## Data Availability

The datasets analyzed during the current study are available from the corresponding author on reasonable request.

## Abbreviations

ABPS: Aberdeen Back Pain Scale
BDI: Beck Depression Inventory
BPI: Brief Pain Inventory
CPMP: Chronic primary musculoskeletal pain
CPP: Chronic primary pain
DSM-III, III-R, IV, 5: Diagnostic and Statistical Manual of Mental Disorders, 3rd edition (1980), 3rd edition—revised (1987), 4th edition (1994), 5th edition (2013)
*E*: Number of events
EQ-5D: EuroQol 5 Dimension
HADS: Hospital Anxiety and Depression Scale
HRQOL: Health-related quality of life
IASP: International Association for the Study of Pain
ICD-11: International Classification of Diseases, 11th revision
IMMPACT: Initiative on Methods, Measurement, and Pain Assessment in Clinical Trial
MPI: Multidimensional Pain Inventory
Ms: Means
*N*: Total number for each trial arm
NMA: Network meta-analysis
NRS: Numeric rating scale
NSAIDs: Non-steroidal anti-inflammatory drugs
OR: Odds ratio
PGIC: Patient Global Impression of Change scale
POMS: Profile of Mood States
RCT: Randomized controlled trial
SAEs: Serious adverse events
SDs: Standard deviations
SEs: Standard error
SF-MPQ: Short-Form McGill Pain Questionnaire
SMD: Standardized mean differences
TE: Standardized treatment effect
TEAEs: Treatment-emergent adverse events
UEs: Unwanted events
UME: Unrelated mean effects model
VAS: Visual analog scale
